# Chronic disease management program applied to type 2 diabetes patients and prevention of diabetic complications: a retrospective cohort study using nationwide data

**DOI:** 10.1186/s12889-023-15763-z

**Published:** 2023-05-23

**Authors:** Min Kyung Hyun, Jang Won Lee, Seung-Hyun Ko

**Affiliations:** 1 Department of Preventive Medicine, College of Korean Medicine, Dongguk University, Gyeongju, Republic of Korea; 2 Department of Internal Medicine, Division of Endocrinology and Metabolism, St. Vincent’s Hospital, College of Medicine, The Catholic University of Korea, Seoul, Republic of Korea

**Keywords:** Type 2 diabetes mellitus, Chronic disease management program, Retrospective studies, Diabetes complications, Health education

## Abstract

**Background:**

The outcomes of education and counseling by medical professionals for patients with type 2 diabetes mellitus (T2DM) are unclear. This study examined the effects of the Chronic Disease Management Program (CDMP), a health insurance fee-for-service benefit, on the incidence of diabetic complications in patients newly diagnosed with T2DM using the National Health Insurance data.

**Methods:**

Patients newly diagnosed with T2DM aged ≥ 20 years from 2010 to 2014 were followed up until 2015. Selection bias was minimized using propensity score matching. A stratified Cox proportional hazards model was used to analyze the association between the CDMP and the risk of incident diabetic complications. Subgroup analysis was performed for patients with high medication adherence, which was indicated by a medication possession ratio (MPR) ≥ 80.

**Results:**

Among the 11,915 patients with T2DM in the cohort, 4,617 were assigned to the CDMP and non-CDMP group each. The CDMP helped reduce the overall and microvascular risks of complications compared to the non-CDMP group; however, the protective effect against macrovascular complications was only observed in those aged ≥ 40 years. Subgroup analysis of the group aged ≥ 40 years with high adherence (an MPR ≥ 80) showed that the CDMP effectively reduced the incidence of micro- and macrovascular complications.

**Conclusions:**

Effective management of T2DM is crucial in preventing complications in patients with the condition, and includes regular monitoring and adjustment of treatment by qualified physicians. Nevertheless, long-term prospective studies on the effects of CDMP are required to confirm this finding.

**Supplementary Information:**

The online version contains supplementary material available at 10.1186/s12889-023-15763-z.

## Introduction

Globally, type 2 diabetes mellitus (T2DM) and its complications have a significant economic impact on both individuals and nations. The International Diabetes Federation Diabetes Atlas estimates that the global direct health expenditure on diabetes mellitus (DM) will reach $1.03 trillion by 2030 and $1.05 trillion by 2045 [1]. According to the Diabetes Fact Sheet in Korea from the Korean Diabetes Society, 13.8% and 28% of adults aged ≥ 30 and ≥ 65 years, respectively, had diabetes in 2018 [2]. Moreover, the prevalence of T2DM among individuals under 30 years of age increased 4.43-fold between 2002 and 2016 and was particularly high among adolescents aged 10–19 years from low-income families [3].

An increasing number of patients with diabetes experience complications. A multinational observational study showed that 53.5% and 27.0% of patients with T2DM present with microvascular and macrovascular complications, respectively [4]. According to a study that utilized a T2DM simulation model to estimate the lifetime direct medical costs for patients newly diagnosed with T2DM in the United States, 53% of the total expenses for T2DM treatment were allocated to managing diabetic complications; macrovascular complication management costs accounted for 57% of the total complication costs [5]. In addition, a study analyzing the French National Health Insurance Administrative Database and the French SHI database (Système National des données de santé or SNDS) reported that the excess costs associated with hospitalization of patients with T2DM from 2006 to 2015 could be related to diabetic complications [6]. In addition to diabetic vascular complications, certain critical conditions, such as infection, severe hypoglycemia, or acute metabolic decompensation, typically necessitate hospitalization in patients with diabetes, leading to increased medical expenses.

Consistent glycemic control within the target range and lifestyle modifications, such as maintaining a healthy body weight, eating a healthy diet, being physically active, abstaining from smoking, and consuming alcohol in moderation have been demonstrated to decrease the incidence of diabetic complications [7]. Additionally, diabetes education by medical personnel can promote medication adherence and healthy lifestyle maintenance, thereby reducing the likelihood of developing complications [8]. Contrarily, health insurance premiums prioritizes screening, medications, and procedures over education and counseling due to insufficient evidence regarding the latter’s efficacy. Medical personnel are familiar with the related medical tests conducted in clinics, as well as understand patients' lifestyles outside clinics [9, 10]. A consensus on the importance of lifestyle modifications in patients with diabetes was established and nationwide efforts were initiated in the Republic of Korea (ROK) in 2012. A multilevel intervention, including copayment reduction and physician incentives, called the Chronic Disease Management Program (CDMP), was introduced in 2012 to improve blood pressure and glycemic control by strengthening the function of clinics as primary care institutions for managing hypertension and diabetes [11]. Hypertension management using the CDMP has proven to be highly cost-effective in patients with hypertension aged ≥ 40 years [12]. However, the clinical- and cost effectiveness of the CDMP in managing diabetes has not been clarified. Therefore, this study aimed to determine the clinical effectiveness of CDMP implementation in preventing diabetic complications among patients newly diagnosed with T2DM by analyzing the National Health Insurance (NHI) data. We hypothesized that, as in the case of hypertension, similar clinical effectiveness would be observed for diabetes.

## Methods

### Study design and data source

**Fig. 1:**
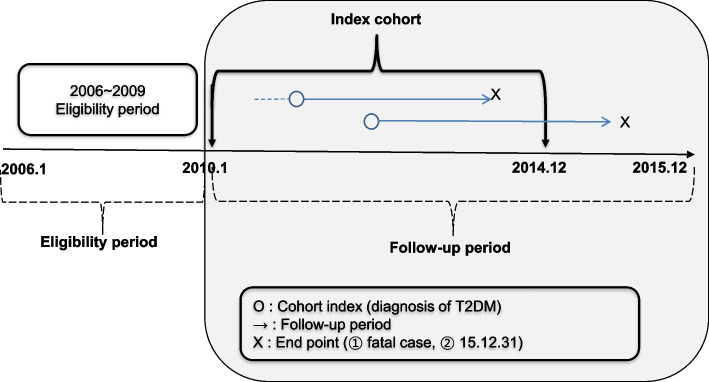
Study design. Abbreviations: T2DM, Type 2 Diabetes mellitus

This retrospective cohort study used an index date from January 2010 to December 2014, with an eligibility period spanning from January 2006 to December 2009. The final cohort was followed up until December 2015 (Fig. 1).

This study was conducted using the National Health Insurance Service–National Sample Cohort (NHIS–NSC), which consists of representative anonymized data extracted from the NHI records of approximately 1,000,000 participants from 2002 to 2015 [13]. The NHI is a compulsory social health insurance program run by the ROK government that covers approximately 97% of the population living in the ROK, with the exception of those enrolled in the medical aid program.

### Participants

The inclusion criteria for the patient cohort were patients aged ≥ 20 years newly diagnosed with T2DM between 2014 and 2016. T2DM was diagnosed using the E11, E13, and E14 codes based on the Korean Standard Classification of Diseases (KCD), which is the same as the 10^th^ revision of the International Classification of Diseases (ICD-10).

**Fig. 2:**
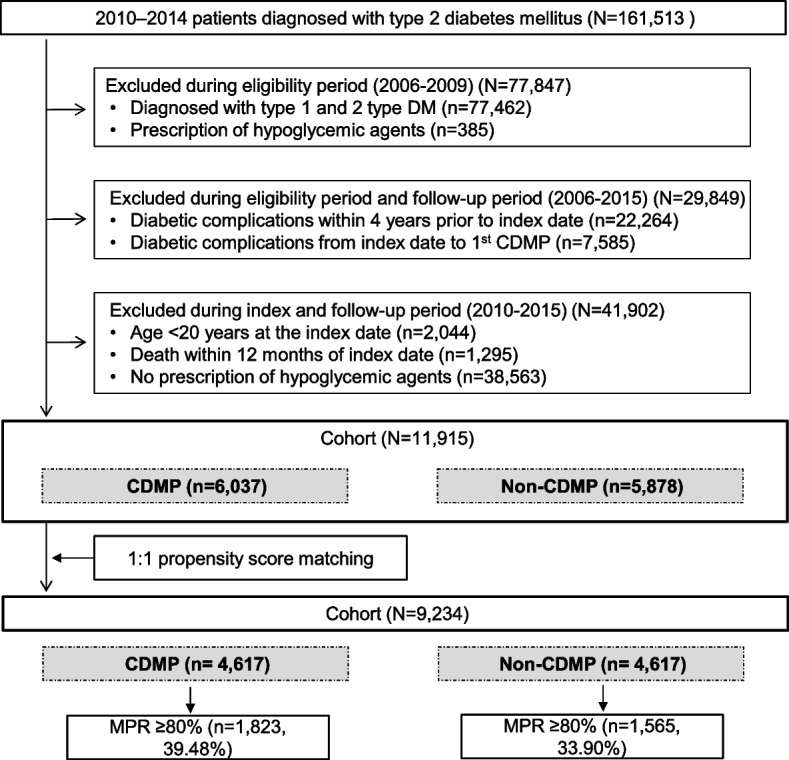
Participant selection. Abbreviations: CDMP, Chronic Disease Management Program; DM, Diabetes mellitus; MRP, Medication possession ratio

Patients were excluded based on the following criteria:

1. Patients diagnosed with type 1 or type 2 DM or those prescribed hypoglycemic agents during the eligibility period.
2. Patients who had diabetic complications in the four years prior to the index date or prior to receiving the first CDMP.
3. Patients under 20 years of age or with a follow-up period of less than one year due to death one year after diagnosis or without a prescription for hypoglycemic agents (Fig. 2).

### Variable definitions

#### Chronic disease management program

The CDMP states that the National Health Insurance Service (NHIS) will pay benefits to clinics if medical professionals provide diabetes education and counseling to patients with DM visiting outpatient clinics, with the aim of promoting patient understanding of the disease and to prevent complications. To enhance a patient’s awareness of chronic diseases, medical professionals affiliated with clinics can provide them with patient management handbooks and record disease progress during visits based on the patient’s preference or physician’s discretion.

Each clinic can make a claim to the NHIS up to 12 times a year and up to twice a month for one patient.

#### Antidiabetic medication and diabetes complications

A draft of the codebook for antidiabetic medication and diabetes complications was prepared after reviewing the clinical practice guidelines and previous studies [14–17]. Subsequently, the final lists were completed after an independent review by three endocrinologists treating patients with T2DM at clinics regarding the completeness of the codebook and their general reflections of actual clinical practice. Antidiabetic medications were classified as metformin, sulphonylureas, alpha-glucosidase inhibitors, meglitinides, thiazolidinediones, dipeptidyl peptidase-4 inhibitors, sodium-glucose cotransporter-2 inhibitors, glucagon-like peptide-1 receptor agonists, and insulin (Table S1). Insulin was classified as follows: rapid-acting insulin, short-acting or intermediate-acting insulin, long-acting insulin, and premixed insulin (Table S1).

The complications of T2DM have traditionally been divided into macrovascular and microvascular complications [18]. Microvascular complications include retinopathy, blindness, nephropathy, chronic renal failure, end-stage renal disease, and neuropathy. Macrovascular complications included foot ulcers, ischemic heart disease/myocardial infarction, ischemic stroke, hemorrhagic stroke, and peripheral vascular diseases (Table S2). Complications were considered to have occurred when there was a history of 1) three or more outpatient visits; 2) one or more hospitalizations; and 3) One or more procedures related to diabetic complications performed after diagnosis of T2DM.

#### Medication adherence

Medication adherence was measured using the medication possession ratio (MPR), one of the most widely used indirect measurement indices [19]. The MPR was calculated by dividing the total days of medication supplied (excluding the days supplied for the last clinic visit) by the number of days between the first and last refills [20]. An MPR ≥ 80% was considered high adherence.

### Statistical analysis

Descriptive statistical analyses were performed to characterize study participants. A 1:1 case–control match was performed on the propensity score (nearest-neighbor matching). Propensity score matching (PSM) is commonly used to reduce bias from concomitant confounding variables and correct baseline imbalances [21]. The incidence of diabetes-related complications was compared between the CDMP and non-CDMP groups by using a stratified Cox proportional hazards model. This extended Cox model allows control through the stratification of a predictor that does not satisfy the proportional hazards assumption [22]. To test for proportionality, time-dependent covariates were generated by producing interactions between predictors as functions of survival time; these data were then included in the model. If any time-dependent covariate was significant, the predictors were not proportional. In this study, age group did not satisfy the proportional hazards assumption; therefore, the age group variable was adjusted for by stratification. Other variables were adjusted for inclusion in the model. Age groups were stratified into 20–39, 40–59, and ≥ 60 years. The covariates included in the stratified Cox proportional hazards model were sex, year of diagnosis, economic status, healthcare insurance type, region of residence, hypertension (I10-I13), hyperlipidemia (E78), Charlson Comorbidity Index, follow-up period, and pattern of antidiabetic medication prescription. Subgroup analysis was performed to determine the risk of diabetic complications among patients with high medication adherence (an MPR ≥ 80).

All data manipulations and statistical analyses were performed using the SAS software (version 9.4; SAS Institute Inc., Cary, NC, USA). A *p*-value of < 0.0001 was considered significant.

## Results

### Characteristics of the participants

The final cohort included 11,915 patients newly diagnosed with T2DM between 2010 and 2014 who met the eligibility criteria. The PSM method adjusted for characteristic variables was applied, and the CDMP and non-CDMP groups each included 4,617 patients. Balance or comparability of the measured pre-treatment covariates was achieved between the groups, controlling for confounding bias when estimating the treatment effects (Table 1).

**Table 1:** Characteristics of the participants before and after propensity score matching

Variables	Before PSM						After PSM					
	**CDMF (*n* = 6,037)**		**Non-CDMF (*n* = 5,878)**		**SMD**	***P*-value**	**CDMP (*n* = 4,617)**		**Non-CDMP (*n* = 4,617)**		**SMD**	***P*-value**
	**n**	**%**	**n**	**%**			**n**	**%**	**n**	**%**		
Age group												
20–39	685	11.35%	935	15.91%	0.1354	< 0.0001	611	13.23%	628	13.60%	0.019	0.6554
40–59	3,558	58.94%	3,346	56.92%			2,716	58.83%	2,736	59.26%		
≥ 60	1,794	29.72%	1,597	27.17%			1,290	27.94%	1,253	27.14%		
Sex												
Male	3,578	59.27%	3,716	63.22%	0.0812	< 0.0001	2,884	62.46%	2,862	61.99%	0.010	0.6368
Female	2,459	40.73%	2,162	36.78%			1,733	37.54%	1,755	38.01%		
Year of diagnosis												
2010	1,264	20.94%	1,147	19.51%	0.0747	0.0023	949	20.55%	927	20.08%	0.023	0.8739
2011	1,316	21.80%	1,186	20.18%			940	20.36%	971	21.03%		
2012	1,229	20.36%	1,189	20.23%			964	20.88%	938	20.32%		
2013	1,178	19.51%	1,196	20.35%			930	20.14%	934	20.23%		
2014	1,050	17.39%	1,160	19.73%			834	18.06%	847	18.35%		
Economic status (quantile)												
1st (low)	885	15.22%	832	14.83%	0.0192	0.9012	680	14.73%	698	15.12%	0.032	0.6781
2nd	906	15.58%	905	16.13%			740	16.03%	734	15.90%		
3rd	1,109	19.07%	1,084	19.32%			847	18.35%	893	19.34%		
4th	1,326	22.80%	1,262	22.49%			1,080	23.39%	1,042	22.57%		
5th (high)	1,590	27.34%	1,529	27.25%			1,270	27.51%	1,250	27.07%		
Healthcare insurance type^a^												
NHIS, employees	2,234	37.01%	2,001	34.04%	0.0683	0.0010	1,696	36.73%	1,711	37.06%	0.007	0.7463
NHIS, self-employed	3,620	59.97%	3,661	62.28%			2,921	63.27%	2,906	62.94%		
Medical aid	182	3.02%	216	3.67%			0	0.00%	0	0.00%		
Region of residence^a^												
Seoul capital area	3,003	49.75%	2,746	46.72%	0.0766	0.0002	2,246	48.65%	2,242	48.56%	0.002	0.9937
Metropolitan city	1,213	20.10%	1,154	19.63%			916	19.84%	915	19.82%		
Other regions	1,820	30.15%	1,978	33.65%			1,455	31.51%	1,460	31.62%		
Hypertension												
No	3,491	57.83%	4,249	72.29%	0.312	< 0.0001	3,152	68.27%	3,152	68.27%	< 0.001	1.000
Yes	2,546	42.17%	1,629	27.71%			1,465	31.73%	1,465	31.73%		
Hyperlipidemia												
No	3,421	56.67%	3,891	66.20%	0.197	< 0.0001	2,932	63.50%	2,926	63.37%	0.003	0.8968
Yes	2,616	43.33%	1,987	33.80%			1,685	36.50%	1,691	36.63%		
Charlson Comorbidity Index												
0	986	16.33%	1,009	17.17%	0.0788	0.0001	799	17.31%	775	16.79%	0.016	0.7327
1–2	2,328	38.56%	2,044	34.77%			1,674	36.26%	1,666	36.08%		
≥ 3	2,723	45.11%	2,825	48.06%			2,144	46.44%	2,176	47.13%		

*Abbreviations*: *CDMP*, Chronic Disease Management Program, *PSM* propensity score matching

^a^At the time of type 2 diabetes diagnosis

### Diabetes medication and complications by group

The classes of antidiabetic medications used, namely metformin, sulfonylureas, thiazolidinediones, dipeptidyl peptidase-4 inhibitors, and insulin, showed significant differences between the groups. Additionally, significant differences were observed between the groups regarding the patterns of antidiabetic medication prescription, MPR, number of outpatient visits, number of complications, and type of complications. An MPR of ≥ 80 was noted in 39.48% and 33.90% of patients in the CDMP and non-CDMP groups, respectively. In contrast, an MPR of ≤ 19 was observed in 20.16% and 37.34% of patients in the CDMP and non-CDMP groups, respectively. In the CDMP group, the incidence of ≥ 15 outpatient visits during the follow-up period was 53.47%, whereas seven or fewer visits were noted in 47.71% of the patients in the non-CDMP group (Table 2).

**Table 2:** Diabetes medication and complications

Variables	CDMP (*n* = 4,617)		Non-CDMP (*n* = 4,617)		*P* value
	**n**	**%**	**n**	**%**	
Follow-up period (year)					
Mean ± SD	3.53 ± 1.41		3.45 ± 1.43		0.0046
Median (IQR)	3.55 (2.35, 4.77)		3.44 (2.19, 4.69)		
Pattern of antidiabetic medication prescription					
Mono	1,737	39.08%	1,793	47.90%	< 0.0001
Dual	1,557	35.03%	1,123	30.00%	
Triple	1,050	23.62%	656	17.53%	
Insulin	101	2.27%	171	4.57%	
Class of antidiabetic medication					
Metformin	4,032	87.33%	3,279	71.02%	< 0.0001
Sulfonylureas	2,098	45.44%	1,424	30.84%	< 0.0001
Alpha-glucosidase inhibitors	167	3.62%	154	3.34%	0.4602
Meglitinides	33	0.71%	58	1.26%	0.0084
Thiazolidinediones	353	7.65%	227	4.92%	< 0.0001
Dipeptidyl peptidase-4 inhibitors	1,679	36.37%	1,220	26.42%	< 0.0001
Sodium-glucose cotransporter-2 inhibitors	98	2.12%	77	1.67%	0.1090
Glucagon-like peptide-1 receptor agonists	1	0.02%	2	0.04%	0.5636
Insulin	101	2.19%	171	3.70%	< 0.0001
Rapid-acting insulin	5	.011%	11	0.24%	0.1333
Short-acting insulin or Intermediate-acting insulin	11	0.24%	33	0.71%	0.0009
Long-acting insulin	67	1.45%	106	2.30%	0.0028
Premixed insulin	2	0.04%	2	0.04%	1.0000
Medication adherence (medication possession ratio)					
0–19	931	20.16%	1,724	37.34%	< 0.0001
20–39	526	11.39%	381	8.25%	
40–59	588	12.74%	372	8.06%	
60–79	749	16.22%	575	12.45%	
80–100	1,823	39.48%	1,565	33.90%	
Length of hospitalization^a^					
Mean ± SD	1.72 ± 2.44		2.36 ± 4.77		0.0619
Median (IQR)	1 (1, 1)		1 (1, 2)		
0	4,487	97.18%	4,286	92.83%	< 0.0001
1 ≤	130	2.82%	331	7.17%	
Number of outpatient visits^a^					
Mean ± SD	20.45 ± 18.30		12.96 ± 15.02		< 0.0001
Median (IQR)	16 (6, 30)		8 (2, 19)		
0 ~ 7	1,207	29.67%	1,565	47.71%	< 0.0001
8–14	686	16.86%	621	18.93%	
15 ≤	2,175	53.47%	1,094	33.35%	
Number of complications					
Mean ± SD	0.02 ± 0.14		0.19 ± 0.48		< 0.0001
Type of complications					
Microvascular complications	45	0.97%	385	8.34%	< 0.0001
Retinopathy	15	0.32%	135	2.92%	< 0.0001
Blindness	0	0.00%	1	0.02%	0.3173
Nephropathy	10	0.22%	63	1.36%	< 0.0001
Chronic kidney failure	0	0.00%	43	0.93%	< 0.0001
End-stage renal disease	1	0.02%	36	0.78%	< 0.0001
Neuropathy	21	0.45%	183	3.96%	< 0.0001
Macrovascular complications	28	0.61%	376	8.14%	< 0.0001
Foot ulcer	14	0.30%	70	1.52%	< 0.0001
Ischemic heart disease, myocardial infarction	7	0.15%	158	3.42%	< 0.0001
Ischemic stroke	3	0.06%	91	1.97%	< 0.0001
Hemorrhagic stroke	0	0.00%	40	0.87%	< 0.0001
Peripheral vascular disease	4	0.09%	40	0.87%	< 0.0001

*Abbreviations*: *CDMP* Chronic Disease Management Program

^a^Due to diabetes mellitus during follow-up period

### Risk assessment of diabetic complications by group

**Fig. 3:**
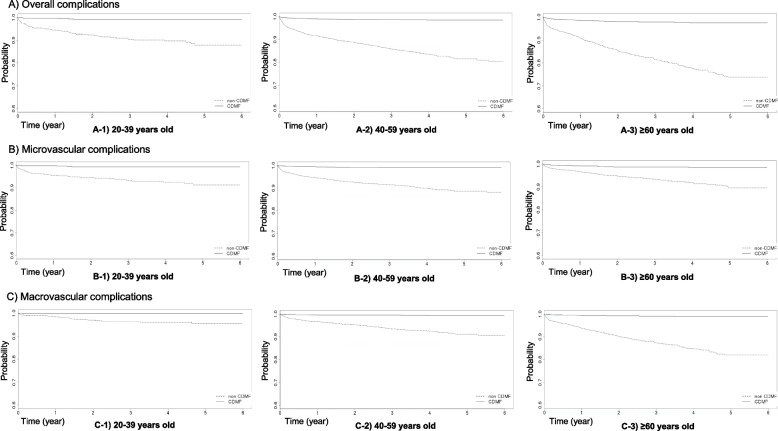
Kaplan‒Meier estimates of incidence of diabetic complications by age group

The CDMP helped reduce the overall risk of complications, including microvascular and macrovascular complications, compared with the non-CDMF group (HR 0.079, 95% CI, 0.061–0.102). It also reduced the risk of overall and microvascular complications in all age groups. In contrast, the CDMP reduced the risk of macrovascular complications only in the 40–59-year-old and ≥ 60-year-old groups (Table 3, Fig. 3).

**Table 3:** Multivariate hazard ratios of the incidence of complications according to the age group

Outcomes	Total (*n* = 9,234)				20–39 years old (*n* = 1,239)				40–59 years old (*n* = 5,452)				≥ 60 years old (*n* = 2,543)			
	**n (%) of Events**	**HR**^a^	**95% CI**	***P* value**	**n (%) of Events**	**HR**^a^	**95% CI**	***P* value**	**n (%) of Events**	**HR**^a^	**95% CI**	***P* value**	**n (%) of Events**	**HR**^a^	**95% CI**	***P* value**
**Overall complications**																
CDMP																
No	710 (15.38)	**Ref**		< 0.0001	60 (9.55)	**Ref**		< 0.0001	407 (14.88)	**Ref**		< 0.0001	243 (19.39)	**Ref**		< 0.0001
Yes	70 (1.52)	0.079	0.061–0.102		5 (0.82)	0.053	0.021–0.133		37 (1.36)	0.075	0.054–0.106		28 (2.17)	0.090	0.059–0.138	
**Microvascular complications**																
CDMP																
No	385 (8.34)	**Ref**		< 0.0001	43 (6.85)	**Ref**		< 0.0001	251 (9.17)	**Ref**		< 0.0001	91 (7.26)	**Ref**		< 0.0001
Yes	45 (0.97)	0.092	0.067–0.127		4 (0.65)	0.058	0.020–0.164		24 (0.88)	0.079	0.051–0.121		17 (1.32)	0.136	0.079–0.235	
**Macrovascular complications**																
CDMP																
No	376 (8.14)	**Ref**		< 0.0001	23 (3.66)	**Ref**		0.0011	187 (6.83)	**Ref**		< 0.0001	166 (13.25)	**Ref**		< 0.0001
Yes	28 (0.61)	0.067	0.045–0.100		1 (0.16)	0.034	0.004–0.256		15 (0.55)	0.076	0.045–0.129		12 (0.93)	0.061	0.032–0.116	

*Abbreviations*: *CDMP* Chronic Disease Management Program, *HR* hazard ratio, *CI* confidence interval

^a^Adjusted for age group (all-subject analyses only), sex, year of diagnosis, economic status, healthcare insurance type, region of residence, hypertension, hyperlipidemia, Charlson Comorbidity Index, follow-up period, and pattern of antidiabetic medication prescription

### Subgroup analysis

When analyzing patients with high medication adherence (an MPR ≥ 80), the CDMP reduced the risk of overall complications (HR 0.060, 95% CI 0.040–0.090). Nevertheless, when divided according to the age group and complication type, the reduction in the risk of microvascular and macrovascular complications by the CDMP was effective only in the ≥ 40-year-old group (Table 4).

**Table 4:** Subgroup analysis of patients with high medication adherence (medication possession ratio ≥ 80)

Outcomes	Total (*n* = 3,388)				20–39 years old (*n* = 329)				40–59 years old (*n* = 2,016)				over 60 years old (*n* = 1,043)			
	**n (%) of Events**	**HR**^a^	**95% CI**	***P* value**	**n (%) of Events**	**HR**^a^	**95% CI**	***P* value**	**n (%) of Events**	**HR**^a^	**95% CI**	***P* value**	**n (%) of Events**	**HR**^a^	**95% CI**	***P* value**
**Overall complications**																
CDMP																
No	310 (19.81)	**Ref**		< 0.0001	28 (18.06)	**Ref**		< 0.0001	**188 (19.69)**	**Ref**		< 0.0001	**94 (20.66)**	**Ref**		< 0.0001
Yes	25 (1.37)	0.060	0.040–0.090		2 (1.15)	0.041	0.009–0.183		13 (1.23)	0.055	0.031–0.096		10 (1.70)	0.070	0.036–0.135	
**Microvascular complications**																
CDMP																
No	202 (12.91)	**Ref**		< 0.0001	22 (14.19)	**Ref**		0.0004	130 (13.61)	**Ref**		< 0.0001	50 (10.99)	**Ref**		< 0.0001
Yes	15 (0.82)	0.059	0.035–0.100		1 (0.57)	0.024	0.003–0.192		7 (0.66)	0.045	0.021–0.097		7 (1.19)	0.106	0.047–0.237	
**Macrovascular complications**																
CDMP																
No	138 (8.82)	**Ref**		< 0.0001	11 (7.10)	**Ref**		0.0246	75 (7.85)	**Ref**		< 0.0001	52 (11.43)	**Ref**		< 0.0001
Yes	11 (0.60)	0.062	0.034–0.115		1 (0.57)	0.085	0.010–0.730		6 (0.57)	0.069	0.030–0.158		4 (0.68)	0.052	0.019–0.145	

*Abbreviations*: *CDMP* Chronic Disease Management Program, *HR* hazard ratio,*CI* confidence interval

^a^Adjusted for age group (all subject analysis only), sex, year of diagnosis, economic status, healthcare insurance type, region of residence, hypertension, hyperlipidemia, Charlson Comorbidity Index, follow-up period, and pattern of antidiabetic medication prescription

## Discussion

The CDMP was effective in reducing the incidence of diabetes-related complications in patients newly diagnosed with T2DM, with a mean follow-up of 3.5 years in the CDMP group and 3.45 years in the non-CDMP group. The present study found that, similar to the cost-effectiveness of the CDMP for hypertension in individuals aged ≥ 40 years, the prevention of complications associated with type 2 diabetes was more evident in those aged ≥ 40 years [12]. Prevention and management of complications after the onset of T2DM are crucial in terms of personal and national health expenditures. A recent NHI data analysis confirmed that the high cost of diabetes is primarily driven by diabetic complications or related comorbidities and hospitalization, as shown by the annual diabetes cost estimation study using the Korean Health Insurance Review and Assessment Service National Patient Sample data [23]. Additionally, a NHIS-NSC data analysis showed that the annual prevalence of diabetic neuropathy decreased from 24.9% in 2006 to 20.8% in 2015, although the underlying cause for this decrease was unclear [24]. This reduction may be attributed to the decrease in proliferative diabetic retinopathy from 1.29% in 2006 to 1.16% in 2015, which in turn is partly attributed to the early diagnosis of diabetic retinopathy, as well as improved glycemic control achieved through new antiglycemic agents and appropriate treatment [25]. In addition, the rate of hospitalization due to major cardiovascular complications has also decreased [26]. The reasons for the significant reduction in these complications have not been confirmed; however, effective management of patients with diabetes is believed to be a contributing factor. These results also confirm the hypothesis that patient education and counseling by medical professionals for preventing complications in T2DM patients have a significant and beneficial effect on the frequency of T2DM complications. Theoretically, the CDMP can improve medication compliance and optimize prescriptions through closer monitoring. Indeed, better medication compliance was observed in the CDMP group, even among patients with multiple prescriptions. This finding is supported by the higher frequency of outpatient visits among CDMP participants. Under Japan's universal public health insurance system, a five-year follow-up of local disease management programs, including patient education programs with a focus on preventing diabetic complications, showed that these programs were somewhat effective in reducing diabetic complications and the need for emergency care [27]. In a retrospective chart review conducted in the United States and published in 2022, the effects of comprehensive diabetes education on the reduction of glycated hemoglobin (HbA1c) and fasting blood glucose were reported [8]. This initiative included explanations of the importance of diet, exercise, medication use, annual eye examinations, hyperglycemia, and HbA1c testing [8].

Recent studies have reported that coach-facilitated, technology-assisted diabetes self-management education can help patients with T2DM manage their disease [28–31]. Smart healthcare is an intelligent service that enables more efficient, convenient, and personalized treatment by monitoring and managing individual health status in real time by combining healthcare with digital technologies, such as big data, artificial intelligence, the Internet of Things, and cloud computing [32]. Despite the widespread use of smart healthcare, there is a lack of evidence-based research to support its efficacy in T2DM management. Evidence should be generated simultaneously with the verification of T2DM education effectiveness in actual clinical practice. These research directions will pave the way for the development of more effective strategies to improve T2DM outcomes.

The ROK has a well-functioning and cost-effective healthcare system, as evidenced by health achievements confirmed in national and international health statistics and risk management during the recent COVID-19 pandemic [33, 34]. Despite this, the average patient consultation time is approximately six minutes, which is shorter than the patient's expectations. The lack of in-depth consultations is a problem [35, 36]. A study published in 2018 suggested that the ROK healthcare system should be reorganized to include preventive and rehabilitation services to care for elderly individuals with chronic diseases and help resolve their unmet healthcare needs [37]. In 2020, public medical institutions accounted for 5.4% of all medical institutions in the ROK, the lowest among the OECD countries [38]. This is also related to the uniquely fragmented healthcare system in which public health centers are in charge of public health, specifically disease prevention, whereas private medical institutions are in charge of disease treatments. However, with the revision of the ‘Public Health and Medical Services Act’ in 2012, the definition of public health has been redefined from describing the ownership of a medical institution performing public health to the function of a medical institution. Now, private medical institutions also perform public healthcare functions [39]. In recent years, various healthcare pilot projects, such as a pilot project supporting the discharge of acute-stage patients and linking activities with the local community, a pilot project aimed at helping primary care physicians care for the disabled, and a pilot project for primary care chronic disease management, have been carried out to improve the healthcare system to provide integrated support, including prevention, treatment, and even improved welfare [40, 41]. Although health policy experts are somewhat disinterested, a claim can be made to the NHIS if a physician provides education and counseling to patients with diabetes even under the current health insurance system in the ROK. Despite the time and effort required for education and counseling, health insurance premiums are relatively inexpensive in the ROK compared to those of other countries with similar purchasing power. Therefore, medical institutions tend to focus on medical device-based tests and prescription drugs for profitability [42, 43].

This study had some limitations that should be noted when interpreting the results. First, the CDMP content is determined by individual endocrinologists, and there are no data on patient education materials provided at each visit in the NHIS-NSC database. However, the Korean Diabetes Association provides its member endocrinologists with the latest CPSs, standard patient educational materials, and continuing education. Therefore, endocrinologists’ education levels would have been standardized [14]. Second, the mean follow-up period was short, at 3.53 years for the CDMP group and 3.45 years for the non-CDMP group; hence, it is unclear if the period was sufficient to observe the incidence of complications. The eligibility period to define newly developed T2DM was four years, and it was impossible to extend the observation period because of limited data availability. Further studies using long-term data are required to confirm the findings of this study. Third, this study was conducted on a relatively small number of patients with T2DM compared with to the total number of patients with diabetes because a large number of patients were excluded during the participant selection process. A large-scale prospective cohort study will help to generate more concrete evidence in the future. Evaluating the effectiveness of CDMP in patients younger than 20 years of age and those with type 1 DM is also necessary, given the increased incidence and long-term clinical outcomes of young-onset DM. Fourth, this study used the NHIS-NSC database, which contains secondary data extracted from data collected for NHI administration purposes. Therefore, it is only possible to know whether a blood test has been performed and not the actual results, including blood sugar and HbA1c levels. Despite these limitations, certain beneficial effects of the CDMP have been confirmed. Therefore, additional research is needed to strengthen the evidence for education and counseling for patients with T2DM and to calculate appropriate health insurance premiums.

## Conclusions

This study provides early proof-of-concept data that supports the effectiveness of the CDMP in reducing the risk of diabetic complications. Furthermore, our findings shows that continuous behavior modification through education and counseling is required to sustain these desirable benefits over the long term. Nevertheless, long-term follow-up studies and prospective research are needed to validate these observations to achieve better diabetic management outcomes in patients with T2DM.

## Supplementary Information

**Additional file 1.**

## Data Availability

The data that support the findings of this study are available from [The National Health Insurance Sharing Service (NHISS) website of the National Health Insurance Service (NHIS) (https://nhiss.nhis.or.kr/bd/ab/bdaba021eng.do)]. However, restrictions apply to the availability of these data, which were used under license for the current study, and hence are not publicly available. Upon request, the corresponding author will provide details regarding any restrictions or conditions under which access to certain data may be granted.

## Abbreviations

CDMP: Chronic Disease Management Program
CI: Confidence interval
DM: Diabetes mellitus
HbA1c: Glycated hemoglobin
HR: Hazard ratio
ICD-10: 10^Th^ revision of the International Classification of Diseases
KCD: Korean Standard Classification of Diseases
MPR: Medication Possession Ratio
NHI: National Health Insurance
NHIS: National Health Insurance Service
NHIS-NSC: National Health Insurance Service–National Sample Cohort
PSM: Propensity score matching
ROK: Republic of Korea
T2DM: Type 2 diabetes mellitus
