# Supplementary material for: Chronic disease management program applied to type 2 diabetes patients and prevention of diabetic complications: a retrospective cohort study using nationwide data
Source: BMC Public Health. 2023 May 23;23:928. doi: 10.1186/s12889-023-15763-z (PMC10203667; doi:10.1186/s12889-023-15763-z)
Supplement: Supplementary file 1 — Additional file 1. [file 12889_2023_15763_MOESM1_ESM.docx]

# Table S1. Classes of anti-diabetic medications prescribed

| **Class** | **Compounds** |  |
| --- | --- | --- |
| Metformin | Metformin |  |
| Sulfonylureas | Gliclazide |  |
|  | Glipizide |  |
|  | Glimepride |  |
|  | Glibenclamide |  |
| Alpha-glucosidase inhibitors | Acarbose |  |
|  | Voglibose |  |
|  | Miglitol |  |
| Meglitinides | Repaglinide |  |
|  | Nateglinide |  |
|  | Mitiglinide |  |
| Thiazolidinediones | Piolglitazone |  |
|  | Lobeglitazone |  |
|  | Rosiglitazone |  |
| Dipeptidyl peptidase-4 inhibitors | Sitagliptin |  |
|  | Vildagliptin |  |
|  | Saxagliptin |  |
|  | Linagliptin |  |
|  | Gemigliptin |  |
|  | Alogliptin |  |
|  | Teneligliptin |  |
|  | Anagliptin |  |
|  | Evogliptin |  |
| Sodium-glucose cotransporter-2 inhibitors | Dapagliflozin |  |
|  | Ipragliflozin |  |
|  | Empagliflozin |  |
|  | Ertugliflozin |  |
| Glucagon-like peptide-1 receptor agonists | Exenatide |  |
|  | Lixisenatide |  |
|  | Liraglutide |  |
|  | Dulaglutide |  |
| Insulin | Rapid-acting insulin | Insulin aspart |
|  |  | Insulin glulisine |
|  |  | Insulin lispro |
|  | Short-acting insulin or  Intermediate-acting insulin | Human regular,  Human NPH |
|  | Long-acting insulin | Insulin glargine |
|  |  | Insulin detemir |
|  |  | Insulin degludec |
|  | Premixed insulin | Insulin aspart 70/30 |
|  |  | Insulin aspart 50/50 |
|  |  | Insulin degludec/aspart 70/30 |
|  |  | Insulin lispro 75/25 |
|  |  | Insulin lispro 50/50 |
|  |  | Human NPH/regular 70/30 |

# Table S2. Type of complications of diabetes

|  | | **KCD code** |
| --- | --- | --- |
| Microvascular complication | |  |
|  | Retinopathy | E11.3, E12.3, E13.3. E14.3, H28.0, H35.8, H36.0 |
|  | Blindness | H54 |
|  | Nephropathy | E112, E122, E132, E142 |
|  | Chronic kidney disease | N08.3, N18, N19 |
|  | End-stage renal disease | N18, Z94.0 |
|  | Neuropathy | E11.4, E12.4, E13.4, E14.4, G59.0, G63.2, G99.0 |
| Macrovascular complications | |  |
|  | Foot ulcer | E11.7, E13.7, E14.7 |
|  | Ischemic heart disease/ myocardial infarction | I20, I21, I22, I23, I24, I25 |
|  | Ischemic stroke | I63 |
|  | Hemorrhagic stroke | I60, I61, I62 |
|  | Peripheral vascular disease | I700, I702, I708, I709, I738, I739 |
